# Stochastic Model for a 4 QAM Transmission Subject to the Epidemic Interference Effect †

**DOI:** 10.3390/e27060642

**Published:** 2025-06-16

**Authors:** Marcelo S. Alencar

**Affiliations:** Programa de Pós-Graduação em Engenharia Elétrica, Universidade Federal da Paraíba, Campus I, Castelo Branco, João Pessoa 58051-900, PB, Brazil; malencar@iecom.org.br

**Keywords:** wireless communications, 4 QAM modulation, stochastic differentiation, interference analysis

## Abstract

This article presents a stochastic model for the effect of interference caused by a sudden increase in the number of users that access a 4 QAM digital communication system. As demonstrated, the rapid increase in the number of users that access the system causes a non-stationary traffic in the network. A stochastic differential approach is used to model the epidemic interference effect.

## 1. Introduction

A mobile telephone system is defined as a communications network via radio that allows continuous mobility by dividing its coverage area into cells. This system suffers from various types of interference, mainly produced by the environment, by different noise sources and by other transmitters that operate in the same frequency range, as discussed in reference [1].

Most problems that have been considered are related to statistically independent interference sources, which generally do not influence each other. Therefore, they are considered independent sources of interference. Therefore, it is usual to assume stationarity in the analysis of the bit error probability [2].

In communication systems, the interference also depends on the state of the network. In the case of a sudden increase in telephone traffic, the usual linear tools, which produce a non-stationary stochastic process, are less useful. Stochastic integration is a new mathematical tool that can be used to attack the problem because it permits dealing with the non-stationary cases.

A non-stationary digital transmission environment is usually complicated in terms of mathematical modelling, therefore it is usual to assume stationarity in the analysis of symbol or bit error probability. In most cases, the assumption is feasible and reliable, and the model works well in real life.

In communication systems, as mentioned, the interference depends on the state of the network, but this is not usually taken into account in the analysis of the systems because of the complexity involved. For instance, if the users access the network in the early morning, the traffic is clearly non-stationary because it is increasing with the number of users that wake up and place calls. The interference also increases as more callers access the telephone system [3].

In the case of a sudden increase in telephone traffic, the usual probability tools, such as basic probabilistic models or stationary stochastic processes, are less useful. However, stochastic integration can be used to attack the problem because it is based on a stochastic differential equation formulation that makes it possible to deal with the non-stationarity of the communications channel. It is important to notice that the subject of non-stationarity, regarding the communications channel, has only recently been addressed [4].

Regarding the contributions, the article presents a stochastic model for the effect of interference caused by a sudden increase in the number of users that access a digital communication system. A demonstration that the rapid increase in the number of users that access the system causes a non-stationary traffic in the network is provided. Different from the previous published papers, a stochastic differential approach is used to model the epidemic interference effect.

In the remainder of the paper, Section 2 presents the concept of instantaneous power interference, which is mathematically modeled in Section 3. Section 4 presents the mathematical results, and a discussion of the obtained results is found in Section 5. Section 6 presents the main conclusions of the paper and future directions of the research.

## 2. The Instantaneous Power Interference

When dealing with complex problems, such as the effect of interference on a mobile communication system, it is usually necessary to compromise, in terms of mathematical modelling. The main reason is that the model could be difficult to verify in practice, or unfeasible to compute in theory.

The stochastic processes that model the interference in a communications system when this system enters an epidemic state, that is, when most users decide to place calls at the same time, are not stationary. This means that their statistical averages, or moments, can vary with time.

The traditional correlation analysis is, thus, not adequate to attack that type of problem. In this case, stochastic differentiation is a useful tool to deal with the situation. This makes it possible to obtain a problem formulation for the modelling of the interference based on a stochastic differential equation.

The stochastic calculus began with the study and modelling of market prices, that is, the fluctuation of the stock value as a function of time. In this case, the investors work based on the variation in the potential gain or loss, dX(t), as a proportion of the invested sum X(t).

Therefore, in fact, what matters is the relative price, dX(t)/X(t), of a certain asset, as it reacts to the market fluctuations, that is, it should be proportional to a Wiener process W(t) [5],

(1) dX(t)=αX(t)dW(t),

which is an informal manner to express the corresponding integral equation [6]

(2) $$X\left(t+\tau \right)-X\left(t\right)=\alpha \int_{t}^{t+\tau }X\left(u\right)dW\left(u\right).$$

In order to model the epidemic effect on the signal transmission, it is important to consider the relative instantaneous power, dP(t)/P(t), of a certain signal as it reacts to the channel interference fluctuations.

As is usually considered in modelling epidemic spreads, the interference that affects the transmitted signal grows at a rate that is proportional to the number of users [7].

The incremental variation of the interference is also proportional to the differential variation dW(t) of a stochastic process, W(t), which usually has a Gaussian distribution and represents the variability of the channel.

Observe that it is multiplied by the total power of the active users, P(t), and should be adjusted by the parameter β, which remains to be found, based on the channel specifications. For instance, β is related to the power exponential growth.

The stochastic process W(t), in turn, is the result of an additive combination of all interference factors, $W_{i}\left(t\right)$, which can be found in a digital communication system. Therefore, by the Central Limit Theorem, that process has a Gaussian probability distribution.

It is natural to combine both preceding assumptions, which results in the following stochastic differential equation [8]:

(3) dP(t)=αP(t)dt+βP(t)dW(t),

in which αP(t) is the drift function, or model trend, and βP(t) is the dispersion function, volatility, of the stochastic process.

The stochastic differential equation obtained with the interference model, can be solved using the generic formulation for stochastic differential equations, derived by Itô.

An immediate question associated with the equation solution is related to the non-differentiability of a Wiener process W(t) at any point in time. A way to circle the problem has been found and is known as the theory of stochastic integrals or the study of stochastic differential equations [9].

## 3. Solution of the Stochastic Equation

Recall that when formulating a stochastic model to represent the reject variation along with time, it is important to consider that the interference rate is proportional to the existing amount of interference. This can be expressed as [7]

(4) $$\frac{dP(t)}{dt}=\alpha P\left(t\right).$$

The incremental variation in the interference is proportional to the differential variation dW(t) of a stochastic process, W(t), which usually has a Gaussian distribution, multiplied by the existing amount of interference, P(t), and adjusted by the parameter β, which remains to be found, based on the channel specifications

(5) dP(t)=βP(t)dW(t).

Combining the previous equations, one obtains (3), which is the informal manner to write

(6) $$P\left(t+\tau \right)-P\left(t\right)=\alpha \int_{t}^{t+\tau }P\left(u\right)du+\beta \int_{t}^{t+\tau }P\left(u\right)dW\left(u\right),$$

in which P(T+τ) represents the future value of the process P(t), which is τ time units in advance.

The solution of the equation follows the procedure established by Itô, which gives

(7) $$P\left(t\right)=P\left(0\right)\cdot e^{\left(\alpha -\frac{\beta^{2}}{2}\right)t+\beta W\left(t\right)}.$$

For the proposed stochastic process, one notes that the interference power grows exponentially in time. On the other hand, it is controlled by the parameters α and β, which remain to be determined based on system and channel conditions. The random variation on the curve is a result of the stochastic process W(t).

The process W(t), which results from *N* independent interference factors caused by the users that access the system, is given by

(8) $$W\left(t\right)=\sum_{i=1}^{N}W_{i}\left(t\right),$$

thus, by the Central Limit Theorem, if *N* is large, W(t) has a Gaussian probability distribution, with mean and variance given by

(9) $$m_{W}\left(t\right)=E\left[W\left(t\right)\right]=\sum_{i=1}^{N}E\left[W_{i}\left(t\right)\right],$$

and

(10) $$\sigma_{W}^{2}\left(t\right)=V\left[W\left(t\right)\right]=\sum_{i=1}^{N}V\left[W_{i}\left(t\right)\right].$$

If its distribution is Gaussian, it is possible to compute the associated interference distribution using the transformation of probability density function mathematical operation. For stationary stochastic processes, or for short instants of time, the moments are independent of time, that is,

(11) $$m_{W}\left(t\right)=Nm_{W}$$

and

(12) $$\sigma_{W}^{2}\left(t\right)=N\sigma_{W}^{2}.$$

Note that the stochastic process, P(t), is non-stationary because its average value depends on time. Additionally, the probability distribution of process P(t), which represents the interference power, is not Gaussian, as is usually assumed in the computation of the error probability using the theory of stochastic processes.

In order to solve the stochastic differential equation obtained with the reject model using the generic formulation for stochastic differential equations, one considered that the drift function is proportional to the stochastic process, αP(t), where the dispersion function is modelled as βP(t), and one can use the Itô formula for the logarithm function f(x,t)=log(x) [6].

Following the procedure established by Itô, for the solution of the equation, one obtains

(13) $$\frac{\partial f(x,t)}{\partial t}=0,$$

(14) $$\frac{\partial f(x,t)}{\partial x}=\frac{1}{x},$$

(15) $$\frac{\partial^{2}f\left(x,t\right)}{\partial^{2}x}=-\frac{1}{x^{2}}.$$

Thus,

(16) $$d\log\left(P\left(t\right)\right)=\frac{\alpha P(t)dt}{P(t)}+\frac{\beta P(t)dW(t)}{P(t)}-\frac{\beta^{2}P^{2}\left(t\right)dt}{2P^{2}\left(t\right)};$$

that is,

(17) $$d\log\left(P\left(t\right)\right)=\left(\alpha -\frac{\beta^{2}}{2}\right)dt+\beta dW\left(t\right).$$

Integrating in time, one obtains

(18) $$\log\left(P\left(t\right)\right)=\log\left(P\left(0\right)\right)+\left(\alpha -\frac{\beta^{2}}{2}\right)t+\beta W\left(t\right),$$

taking the logarithm inverse function, which results in

(19) $$P\left(t\right)=P\left(0\right)\cdot \exp\left(\alpha -\frac{\beta^{2}}{2}\right)t\cdot \exp\left(\beta W\left(t\right)\right),$$

in which P(0) represents the initial value of the process, which remains to be found, based on the problem constraints.

The expected value of the process, P(t), is given by

(20) $$\begin{array}{ccc} E[P(t)] & = & E\left[P\left(0\right)\right]\cdot \exp\left(\alpha -\frac{\beta^{2}}{2}\right)t\cdot E\left[\exp\left(\beta W\left(t\right)\right)\right]\\ & = & E\left[P\left(0\right)\right]e^{\alpha t}. \end{array}$$

Therefore, according to the obtained mathematical model, the average value of the interfering power grows exponentially for the specified conditions. The initial mean value of the process, E[P(0)], should be determined from the data obtained from the received signal, as well as the estimate of the parameter, α, that controls the curve.

### The Influence of the Combined Interference

As discussed in the previous section, the stochastic process W(t) results from the combination of several interferers, $W_{i}\left(t\right)$, as the users decide to place calls at the same time or in the same time interval.

Equation (7) can be simplified if one considers that the system parameters compensate to avoid the exponential growth, that is, α=β/2, which can be carried out by the automatic gain control (AGC) subsystem. The remaining part of the equation is a transformation of probability

(21) $$P\left(t\right)=P\left(0\right)\cdot e^{\beta W(t)}.$$

Considering that W(t) is a Gaussian process, the transformation given by Equation (21) can be solved, giving the probability distribution of the interfering power, P(t) [10]:

(22) $$f_{P}\left(p\right)=\frac{f_{W}\left(w\right)}{\left|dp/dw\right|},\;\;w=f^{-1}\left(p\right);$$

therefore, computing the inverse of Equation (21), one obtains

(23) $$W\left(t\right)=\frac{1}{\beta }\log\left[\frac{P(t)}{P_{0}}\right]=\frac{1}{\beta }\left[\log P(t)-\log P_{0}\right],$$

which can be written in simplified notation as

(24) $$w=\frac{1}{\beta }\left[\log p-\log P_{0}\right],\;p=P\left(t\right).$$

The derivative of the output stochastic process, as a function of the input process, in a simplified notation, is

$$\frac{dp}{dw}=P\left(0\right)\cdot \beta e^{\beta w}.$$

After the substitution of the inverse function, assuming that β is a positive parameter and considering that the exponential is a positive definite function, one obtains

(25) $$f_{P}\left(p\right)=\frac{P_{0}e^{-\frac{{\left[\ln p-\ln P_{o}\right]}^{2}}{2\beta^{2}\sigma_{W}^{2}}}}{\beta p\sigma_{W}\sqrt{2\pi }},$$

if p>0 and null in case p≤0. In the preceding equation, the following notation is used for short, $P_{0}=P\left(0\right)$.

Therefore, the interfering power has a Lognormal distribution. By a property of the Lognormal probability density function, this also implies that the received signal, X(t), has a Lognormal distribution.

## 4. Obtained Results

From the preceding analysis, it is possible to conclude that the random process that represents the interference in a communication system that suffers from an epidemic attack has a Lognormal probability distribution instead of the usual assumption of a Gaussian distribution.

**Figure 1:**
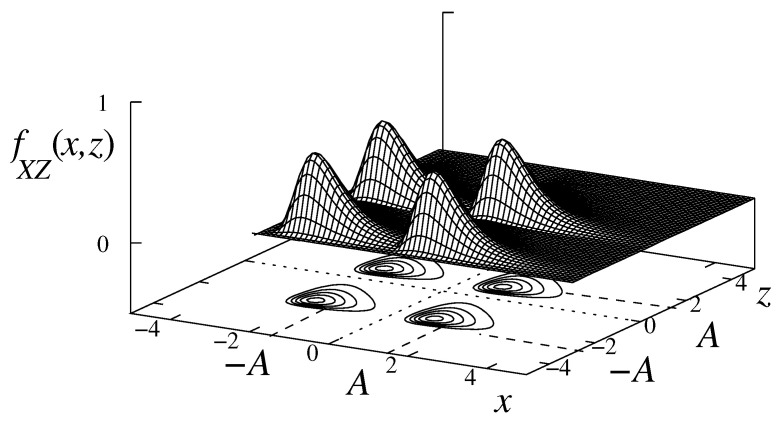
The effect of epidemic interference on the probability distribution, $f_{X,Z}\left(x,z\right)$, of a 4 QAM signal.

This effect is illustrated in Figure 1, which shows the interference is caused by the bivariate Lognormal probability density function (pdf), $f_{X,Z}\left(x,z\right)$, on a 4 QAM signal, with signal symbols in the sets x∈{A,−A} and z∈{A,−A}.

Note that the signal centered at (−A,−A) interferes heavily on the signals centered at (A,−A) and (−A,A), but the contrary is not true. Moreover, the interference on the signal centered at (A,A) can be disregarded on a first approach.

By the same token, the signal centered at (−A,A) interferes heavily with the signals centered on (A,A) and (−A,−A), but the contrary is not true. Finally, they have a smaller interference on the signal centered on (A,−A).

It must also be considered, for the conditions established in the paper, that the interference between the symbols on the *z* axis is weak compared with the interference between the symbols on the *x* axis.

That result can be used to compute the symbol or bit error probability for different modulation schemes. As it is known, the Lognormal distribution has a heavy tail; that is, it decays at a much slower pace than the exponential function.

Therefore, an increase in the error probability during the communication system power adaptation process is thus expected. This could disconnect the call because maintaining an acceptable error rate is one of the conditions to continue the telephone connection.

The bit error rate for a 4 QAM signal, for the case of a zero mean Gaussian distribution, can be obtained by numerically computing the integral

(26) $$P_{E}=\int_{\frac{A}{\sigma }}^{\infty }\frac{1}{\sqrt{2\pi }}e^{-\frac{1}{2}t^{2}}dt,$$

in which *A* is the modulated signal amplitude. The equation can be rewritten using the transformation $t=\frac{x+A}{\sigma }$, dx=σdt to obtain the error probability.

The preceding result can be simplified to

(27) $$P_{E}=Q\left(\frac{A}{\sigma }\right),$$

using the Q(·) function, which puts into evidence the distribution symmetry and the fact that the errors are equiprobable.

The theoretical BER of an ideal 4 QAM system can be put in the following format [11],

(28) $$P_{E}=Q\left(\sqrt{\frac{2E_{b}}{N_{0}}}\right),$$

in which $E_{b}$ is the energy per bit, and $\frac{N_{0}}{2}$ is the two-sided power spectral density of the additive white Gaussian noise channel.

The Lognormal distribution, on the other hand, is asymmetric and its influence on the interference that affects symbols (−A,−A) and (−A,A) is higher when one computes the error probability. Therefore, most of the error probability is caused by this interference, when the transmission power is high.

The influence of the interference that occurs because of the transmission of symbols (−A,−A) and (−A,A) becomes more evident for a low transmission power, because the Lognormal distribution curve is displaced to the left and eventually crosses the coordinate axes. In this case, the error probability suddenly increases, causing a discontinuity in the curve.

When the channel is under the influence of an epidemic interference, the combined error probability associated with the symbol transmission is given by the following simplified formula, which approximates the exact value:

(29) $$P_{E}=\frac{1}{2}\int_{0}^{\infty }\frac{1}{\left(x+A\right)\sigma \sqrt{2\pi }}e^{-{\left(\frac{\ln\left(x+A\right)}{2\sigma }\right)}^{2}}dx$$

The transformation, $X\left(t\right)=\sqrt{P(t)}$, displaces the interference to the origin of the coordinated system, and the transmitted signal has zero mean. Because of the Lognormal distribution asymmetry, the error probabilities are different for each symbol.

**Figure 2:**
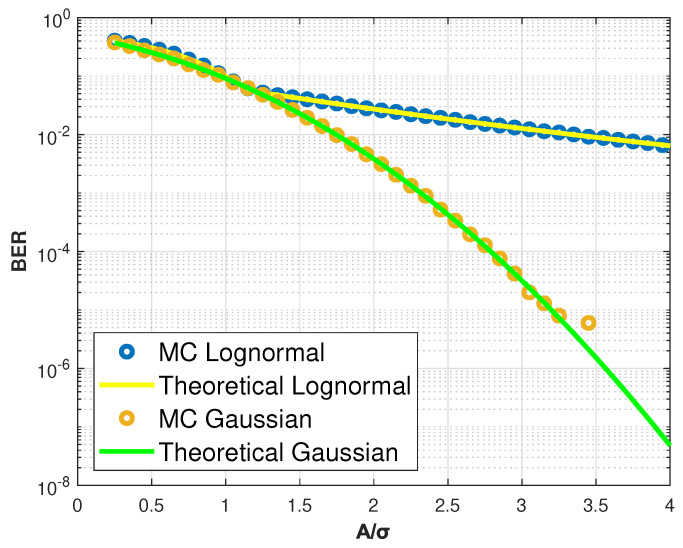
BER as a function of A/σ for 4 QAM modulation.

Monte Carlo simulation has been used to obtain the computation results, which are compared with analytical plots obtained directly from the resulting formulas. The findings are presented in Figure 2, which approximates the results for the 4 QAM modulation.

It is possible to note, for the simulated variance values, that the bit error probability obtained with the Lognormal distribution remains always above that obtained from the Gaussian distribution, which represents the common noise. As can be observed, the Lognormal BER curve decays at a slow rate compared to the Gauss BER plot.

For such a non-stationary process, Itô integration has been used to solve the problem, which, for the defined constraints, resulted in a Lognormal probability density function that, different from the Gaussian distribution, is asymmetrical. The long term interference average and variance values follow exponential curves, which depend on certain system parameters.

## 5. Discussion

Itô integration has been used to solve the problem of stochastic modeling for a 4 QAM transmission subject to the epidemic interference effect. Considering the constraints, the mathematical solution resulted in a Lognormal probability density function that is asymmetrical, in contrast with the Gaussian distribution that is symmetrical for all dimensions. The long term interference average and variance values follow exponential curves, which depend on certain system parameters [12].

## 6. Conclusions

In most analyses, it is usual to assume a stationary channel to compute the symbol or bit error probability, because the system is in a stable state, or to simplify the analysis considering that the non-stationary environment is mathematically complex.

On the other hand, it is well known that the interference is usually dependent on the state of the communications network, for fixed telephony, and also for cellular systems. For instance, if the users decide to access the network all of a sudden, the traffic modelling, as well as the interference, becomes non-stationary.

This article presented a mathematical model for the effect of an interference accumulation, caused by a sudden increase in users in a 4 QAM digital communication system, which is also called an information outbreak or epidemic interference.

This results in a Lognormal distribution for the interference, and for such a heavy tail distribution, there is an expected increase in the error probability during the adaptation process. In the near future, the author plans to develop more complete mathematical and computational models to include *M*-QAM and other modulation formats.

## Data Availability

The original contributions presented in this study are included in the article. Further inquiries can be directed to the author at malencar@iecom.org.br.
